# Microglial activation persists beyond clinical recovery following sport concussion in collegiate athletes

**DOI:** 10.3389/fneur.2023.1127708

**Published:** 2023-03-24

**Authors:** Kiel D. Neumann, Vikram Seshadri, Xavier D. Thompson, Donna K. Broshek, Jason Druzgal, James C. Massey, Benjamin Newman, Jose Reyes, Spenser R. Simpson, Katelyenn S. McCauley, James Patrie, James R. Stone, Bijoy K. Kundu, Jacob E. Resch

**Affiliations:** ^1^Department of Diagnostic Imaging, St. Jude Children's Research Hospital, Memphis, TN, United States; ^2^Department of Radiology and Medical Imaging, University of Virginia, Charlottesville, VA, United States; ^3^Department of Kinesiology, University of Virginia, Charlottesville, VA, United States; ^4^Department of Psychiatry and Neurobehavioral Sciences, University of Virginia, Charlottesville, VA, United States; ^5^Department of Public Health Sciences, University of Virginia, Charlottesville, VA, United States; ^6^Department of Biomedical Engineering, University of Virginia, Charlottesville, VA, United States

**Keywords:** sport concussion, traumatic brain injury, positron emission tomography, neuroinflammation, molecular imaging

## Abstract

**Introduction:**

In concussion, clinical and physiological recovery are increasingly recognized as diverging definitions. This study investigated whether central microglial activation persisted in participants with concussion after receiving an unrestricted return-to-play (uRTP) designation using [^18^F]DPA-714 PET, an *in vivo* marker of microglia activation.

**Methods:**

Eight (5 M, 3 F) current athletes with concussion (Group 1) and 10 (5 M, 5 F) healthy collegiate students (Group 2) were enrolled. Group 1 completed a pre-injury (Visit1) screen, follow-up Visit2 within 24 h of a concussion diagnosis, and Visit3 at the time of uRTP. Healthy participants only completed assessments at Visit2 and Visit3. At Visit2, all participants completed a multidimensional battery of tests followed by a blood draw to determine genotype and study inclusion. At Visit3, participants completed a clinical battery of tests, brain MRI, and brain PET; no imaging tests were performed outside of Visit3.

**Results:**

For Group 1, significant differences were observed between Visits 1 and 2 (*p* < 0.05) in ImPACT, SCAT5 and SOT performance, but not between Visit1 and Visit3 for standard clinical measures (all *p* > 0.05), reflecting clinical recovery. Despite achieving clinical recovery, PET imaging at Visit3 revealed consistently higher [^18^F]DPA-714 tracer distribution volume (VT) of Group 1 compared to Group 2 in 10 brain regions (*p* < 0.001) analyzed from 164 regions of the whole brain, most notably within the limbic system, dorsal striatum, and medial temporal lobe. No notable differences were observed between clinical measures and VT between Group 1 and Group 2 at Visit3.

**Discussion:**

Our study is the first to demonstrate persisting microglial activation in active collegiate athletes who were diagnosed with a sport concussion and cleared for uRTP based on a clinical recovery.

## Introduction

Since 1997, a multidimensional approach has been recommended to diagnose and manage athletes suspected of having a sport concussion (SC) (1, 2). This recommended multidimensional assessment consists of clinical measures of balance, neurocognitive function, and symptoms and has been demonstrated to have a sensitivity of up to 100% within 24 h of a diagnosed SC in collegiate athletes (3, 4). Despite the established clinical utility of this multifaceted approach, a growing body of evidence suggests that physiological recovery from a SC may persist well beyond clinical recovery (5, 6). Although the majority of athletes diagnosed with a SC make an unrestricted return-to-play (uRTP) within 2 weeks of their injury (2), persistent cognitive (7) and motor deficits (8, 9), heightened mood states (10, 11), and other concussion-related symptoms (12) have been observed several months and or years following an unrestricted RTP or retirement as observed in surveyed athletes (11, 13). The etiology of these persisting symptoms remains unclear.

Physiological deficits observed beyond clinical recovery have been primarily reported in high school and collegiate athletes. Wang et al. investigated high school and collegiate football players using standard-of-care clinical measures of SC (6). Participants completed a battery of clinical measures prior to the start of their respective seasons, within 24 h of a diagnosed SC, and 8 days following the injury. Participants also completed a magnetic resonance imaging (MRI) Arterial Spin Labeling protocol to measure cerebral blood flow perfusion. Injured participants were compared to healthy, non-injured football players. Injured participants had significantly lower cerebral blood flow perfusion than the healthy control group at each time point. Despite this finding, the injured group scored within normal limits when compared to pre-injury values on all clinical measures (6). Similarly, Churchill et al. compared collegiate athletes diagnosed with a SC to a healthy collegiate athlete control group using the SCAT3, global functional connectivity, arterial spin labeling, fractional anisotropy, and mean diffusivity. Despite the injured athletes returning to pre-injury levels on the SCAT3 and making an unrestricted RTP, significantly decreased cerebral blood flow and white matter diffusivity were observed up to 1 year following injury (5). Additional research is needed with more sophisticated clinical measures to substantiate the authors' findings (6, 14–16).

The underlying mechanism of the “metabolic crisis” associated with SC has been refined to include a neuroinflammatory response (17). More specifically, the role of microglia following a SC has been questioned largely based on findings in animal models. Microglia play an essential role in surveillance of brain parenchyma and homeostasis (18). Approximately 5–10% of adult brain cells are microglia and are present in all areas of the brain; however, their population density is highly variable (whether under physiological or stress conditions) up to 10-fold in the human brain (19, 20). Microglia function to serve and protect the brain and are wholly dedicated to maintaining homeostasis. Microglia phagocytose apoptotic neurons and secrete inflammatory factors to attract other immune cells to the site of injury. The resolution of this series of events is a tightly regulated process that occurs when injury has been resolved (20, 21). However, if left unresolved, such as the case of chronic or pathologic inflammation, excessive release of cytotoxic factors (reactive oxygen species, pro-inflammatory cytokines, TNF-α, etc.) result in neuronal damage and loss of brain function, which ultimately impede recovery. The persistent over-activation of microglia has been implicated in a number of central nervous system disorders, such as stroke, traumatic brain injury (TBI), mild traumatic brain injury (mTBI), epilepsy, Alzheimer's disease, Parkinson's disease and others (20, 22–24).

Several studies have investigated central neuroinflammation following SC using peripheral fluid biomarkers (25–31). This body of research suggests that neuroinflammation may be short-lived (i.e., < 48 h) and recovery of fluid biomarker levels may occur before clinical recovery as measured by a symptom inventory (4, 32). More recent human-based studies have investigated central inflammation using positron emission tomography (PET) and single-photon emission computerized tomography (SPECT) following a SC (33–35). Originally coined the peripheral benzodiazepine receptor, the translocator protein (TSPO) is an 18 kDa outer mitochondrial membrane protein implicated in a number of neurodegenerative functions including redox homeostasis, modulating immune response, cholesterol transport, and steroidogenesis (36, 37). TSPO expression has been demonstrated to be significantly elevated in microglia as a result of neuronal insult. In addition, the basal expression of TSPO is relatively low (38), thus, TSPO has generated significant interest as an imaging biomarker for microglial activation. Pyrazolopyrimidines DPA-713 and DPA-714 are small molecules with characterized high affinity (Ki = 4.7 nM and Ki = 7.0 nM, respectively) for the TSPO receptor (39, 40).

A recent study investigated [^11^C]DPA-713 binding in former NFL football players whose self-reported number of concussions ranged from 0 to 40 and the span of years since retirement from play ranged from 24 to 42 years. The study revealed significantly higher total distribution volume (VT), as measured by PET, in the hippocampus, amygdala, supramarginal gyrus, and temporal pole compared with healthy age-matched controls (41). A follow up study by Coughlin et al. examined active (*n* = 4) and former (*n* = 10) NFL football players with a range of 1–21 years since their last self-reported concussion. The data revealed significant binding of [^11^C]DPA-713 in the bilateral hippocampus, left entorhinal cortex, bilateral parahippocampal cortices and bilateral supramarginal gyri compared to healthy age-matched controls (33). Higher binding was also independent of age group (active vs. retired play), ethnicity, body mass index, and years of education. Taken together, these studies appear to demonstrate higher microglial activation in NFL players across a wide range of age and participation.

The purpose of this pilot study was to determine the presence of central microglial activation beyond clinical recovery following a diagnosed SC in active, collegiate athletes as compared to healthy collegiate students using TSPO PET. We hypothesized that collegiate athletes diagnosed with a SC would have significantly higher distribution volume (VT) representative of central microglial activation when compared to healthy, physically active, college students. Herein, our findings demonstrate a proof-of-concept that central microglial activation persists beyond clinical recovery in collegiate athletes diagnosed with SC. These findings may be foundational in aligning physiological recovery with clinical recovery.

## Materials and methods

### Study design

Our case-control study design evaluated active and former (one participant is a former collegiate athlete who currently participates in club-level sports) collegiate athletes within 24 h of a diagnosed SC (Group 1). Participants in Group 1 were administered clinical measures of balance, neurocognitive function, and concussion-related symptoms prior to participating in sport [baseline assessment (Visit 1)], at the 24-h post-SC time point (Visit 2), and upon reporting symptom free as well as achieving values consistent with their performance at Visit 1 on each clinical measure (Visit 3). Each visit was in alignment with the NCAA-approved university athletics department concussion management protocol that consists of pre-injury (baseline) and post-injury assessments; however, to enroll in our study and participate in Visit 3, participants consented to obtaining their routine clinical care data, deemed Visit 1 and Visit 2 in our study. These data (Visit 1 and Visit 2) were part of a larger ongoing longitudinal study that consists of pre-injury (baseline) and post-injury testing following a diagnosed concussion (4, 42–44). Therefore, only Group 1 participants who consented to participate in the larger longitudinal study were recruited for this study. Healthy, physically active collegiate students (Group 2) were recruited and evaluated at similar time points as injured participants except for the Visit 1 (i.e., the baseline assessment). In addition to the clinical measures, participants underwent additional neurocognitive testing, functional magnetic resonance imaging, and a PET/CT scan upon achieving clinical recovery. The studies involving human participants were reviewed and approved by the University of Virginia Institutional Review Board and the University of Virginia Radioactive Drug Research Committee. The patients/participants provided their written informed consent to participate in this study. All procedures were performed under the supervision of a licensed physician.

### Participants

Division I collegiate athletes and healthy, active college students were recruited from a large public university to participate in this study. Collegiate athletes were diagnosed with a SC by an athletic trainer or physician. A SC was defined in alignment with the most recent international concussion in sport consensus document (2). Participants were excluded if they had a Glasgow coma scale score < 13, a traumatic injury requiring intensive care unit monitoring or operative repair, a structural abnormality as indicated by computerized tomography, if English was not their primary language, a history of a prior TBI requiring hospitalization, a known brain tumor, a prior self-reported concussion within 3 months of study participation, a known neurologic or neurodevelopmental disorder, a psychiatric disorder requiring hospitalization within 1 year of study participation, an injury resulting from physical abuse (e.g., intimate partner violence), pregnancy, a prior adverse reaction to a radiotracer, if they were a low affinity binder for the TSPO ligand, and/or had an inability to adhere to the study protocol. The same exclusion criteria were applied for control participants. Control participants consisted of healthy, collegiate students who were physically active. Control participants were matched by biological sex and phenotype (e.g., mixed or high affinity) if possible.

### Clinical measures

Participants in Groups 1 and 2 completed the 7-item Generalized Anxiety Disorder (GAD-7), 9-item Patient Health Questionnaire (PHQ-9), Pittsburgh Sleep Quality Index (PSQI), and Sport concussion Assessment Tool [5th Iteration (SCAT5)] (32). In addition to each patient reported outcome measure, each participant was administered; the Immediate Postconcussion Assessment and Cognitive Test (ImPACT) battery (ImPACT Applications, Inc., Pittsburgh, PA) (45), National Institutes of Health (NIH) Toolbox (Reading Recognition test, Dimensional Change Card Sort Test, Flanker Inhibitory Control and Attention Test, and Picture Sequence Memory Test) (46), Rey Auditory Verbal Learning Test (RAVLT) (47), Satisfaction with Life Scale (SWLS) (48), SCAT5 (32), Sensory Organization Test (4), and the Trail Making Test (TMT) Forms A and B (49). All assessments were administered by trained examiners.

Clinical measures were administered at specific visits to decrease the burden on injured athletes and based on previously published evidence of clinical utility (14, 50). For example, participants in Group 1 completed the ImPACT and the SOT at Visits 1–3 as part of routine clinical care and an ongoing longitudinal study related to SC (4, 42–44), while participants in Group 2 completed the ImPACT and SOT at Visit 2. An outline of the protocol may be found in Supplementary Table S1. It is important to note that Visit 3, for some participants, was divided into two testing sessions due to the 5-h protocol length. If Visit 3 was divided into 2 days, neuroimaging would be completed on the first day and the neurocognitive testing would be performed on the second day. Specific to Group 1, in alignment with the University Athletics Department concussion protocol, following a suspected concussion and after reporting symptom-free the athlete was evaluated by an athletic trainer and physician using the most current definition of concussion (2). Upon reporting symptom free, the injured athlete progressed through a standardized graded exercise protocol prior to medical clearance for an unrestricted return to play by a physician.

### DNA extraction and genotyping

Participants in Groups 1 and 2 consented to provide a blood sample for DNA extraction (PureGene^®^ Blood Kit C, Qiagen, Valencia, CA) and TSPO (rs6971) genotyping using the Taqman assay (Applied Biosystems^®^, Life Technologies, Grand Island, NY). Allelic discrimination plots were generated to create standard curves for three distinct TSPO genotypes: Ala147/Ala147 (C/C), Thr147/Thr147 (T/T), and Ala147/Thr147 (C/T). Homozygotes, C/C and T/T, correspond to the high affinity (HAB) and low affinity binding (LAB) phenotype, respectively. The heterozygotes (C/T) correspond to the mixed affinity binding (MAB) phenotype.

### Radiotracer synthesis

[^18^F]DPA-714 was synthesized in accordance with USP < 823> guidelines, as previously described (51). [^18^F]DPA-714 was synthesized in a 13.5 ± 3.1% radiochemical yield (EOB) with a molar activity of 34.3 ± 9.9 Ci/μmol (1,258 ± 366 GBq/μmol) (*n* = 3) in a total synthesis time of 90 min. In all cases, radiochemical purity was >99%. [^18^F]DPA-714 (296 ± 30 MBq) was intravenously injected by bolus injection in high molar activity (1,554 ± 370 GBq/μmol) at the onset of a 90 min dynamic listmode PET acquisition.

### Plasma sampling

Four (4 mL) venous blood samples were collected at 45, 60, 75, and 90 min during the PET scan. Each whole blood sample (1 mL) was counted in a Capintech CRC-55tR to determine whole blood activity in μCi/mL. The remaining whole blood was centrifuged at 13000G for 2 min and plasma was separated and collected into a separate microcentrifuge tube. Approximately 1 mL of acetonitrile was added to the plasma to precipitate serum proteins and the tube was centrifuged at 13000G for 2 min. The remaining supernatant was collected and injected onto a 10 × 250 mm, 10 μm, Gemini C-18 HPLC column (Phenomenex - Torrance, CA). Radioactivity counts were detected using a Posi-RAM high-sensitivity coincidence detector. Radioactive peaks were integrated using Laura PET software (Lablogic – Sheffield, United Kingdom) to determine %parent and %metabolites of [^18^F]DPA-714 in serum. Whole blood samples (1 mL) were separately injected onto a 10 × 250 mm, 10 μm, Gemini C-18 HPLC column and radioactivity counts were detected using a Posi-RAM high-sensitivity coincidence detector to demonstrate no significant differences were measured in %parent_DPA − 714_ of whole blood vs. plasma at these time intervals.

### *In vivo* brain imaging

#### MRI

All subjects underwent a brain MRI for anatomic delineation of Regions of Interest on Positron Emission Tomography images after PET-MRI co-registration. MRI T1-weighted images (256 pixels × 256 pixels × 192 slices) were obtained on a Siemens Prisma 3T scanner with a 64channel head coil using the 3D MP-RAGE sequence as described (52).

#### PET/CT

The same subjects who underwent MRI were imaged for 90 min using the time-of-flight Siemens Biograph mCT (PET-CT) scanner. The dynamic PET data (400 pixels × 400 pixels × 110 slices × 38 time frames) corrected for photon attenuation were generated using an iterative reconstruction algorithm as described (52). The 90-min listmode PET data were binned as follows: [frames, time (s)]: (4 × 7–15 s; 4 × 20–30 s; 3 × 45–60 s; 2 × 90–120 s; 5 × 180–240 s; 12 × 270–300 s).

#### Image analysis

Dynamic imaging was performed with concomitant blood sampling for computing the image derived input function (IDIF) using whole blood radioactivity and correcting for metabolites. PET images, corrected for attenuation and motion, were co-registered with a T1 MR image, gathered immediately prior to the PET scan (52). Briefly, PET data was averaged across the first 14 frames to create a reference used to perform a rigid body transform across the PET frames. The averages of all the motion-corrected PET frames were resliced and co-registered with T1-weighted MRI using non-rigid transform to generate a transformation matrix used, in-turn, to generate a co-registered dynamic PET volume. Next, skull-stripping on the T1-weighted MR image is performed using Brain Extraction Tool (BET) and then deformably registered to the well-known MNI T1-weighted MR brain template (53). The Destrieux atlas (54) parcellation, consisting of a total of 164 regions (82 regions per hemisphere), was simplified to 10 total regions of interest (ROI) based on clinical relevance in human concussion (55–57) and late-stage poor outcomes; these regions included the left and right thalamus, caudate, hippocampus, amygdala, and putamen (58, 59). The 10 regions were also selected based on whole brain deformation models associated with SC and motor vehicle accidents (60). As the deformable registration generates forward and inverse mappings, the simplified Destrieux atlas was propagated to the MR space of each subject. All the above processes were designed using the FMRIB's Software Library (FSL) tool kit (61–65).

To generate the parametric PET maps, a model corrected blood input function (MCIF) was computed by optimizing IDIF derived from the internal carotid arteries using formalism developed for rodent hearts and brains and recently adapted to human brains (52). The dual output model first developed in rodent hearts (66) was further optimized by introducing an additional peak fitting cost function validated in rodent (67) and human brain (52) PET studies. Arterial input is the correct source for deriving the blood input function; however, arterial blood sampling poses many practical challenges. We relied on image derived activity for the first 45 min and then integrated the venous whole blood samples collected at 45, 60, 75 and 90 min during the scan in the computation by introducing a third cost function (tested in rodents) (68), which optimized the MCIF computation, precluding arterial blood sampling. MCIF was further corrected for metabolites by multiplying against a decaying exponential curve (69), which is fitted to values of percent parent blood measured from whole blood samples (70) at four evenly spaced time points from 45 to 90 min during the scan.

**Figure 1:**
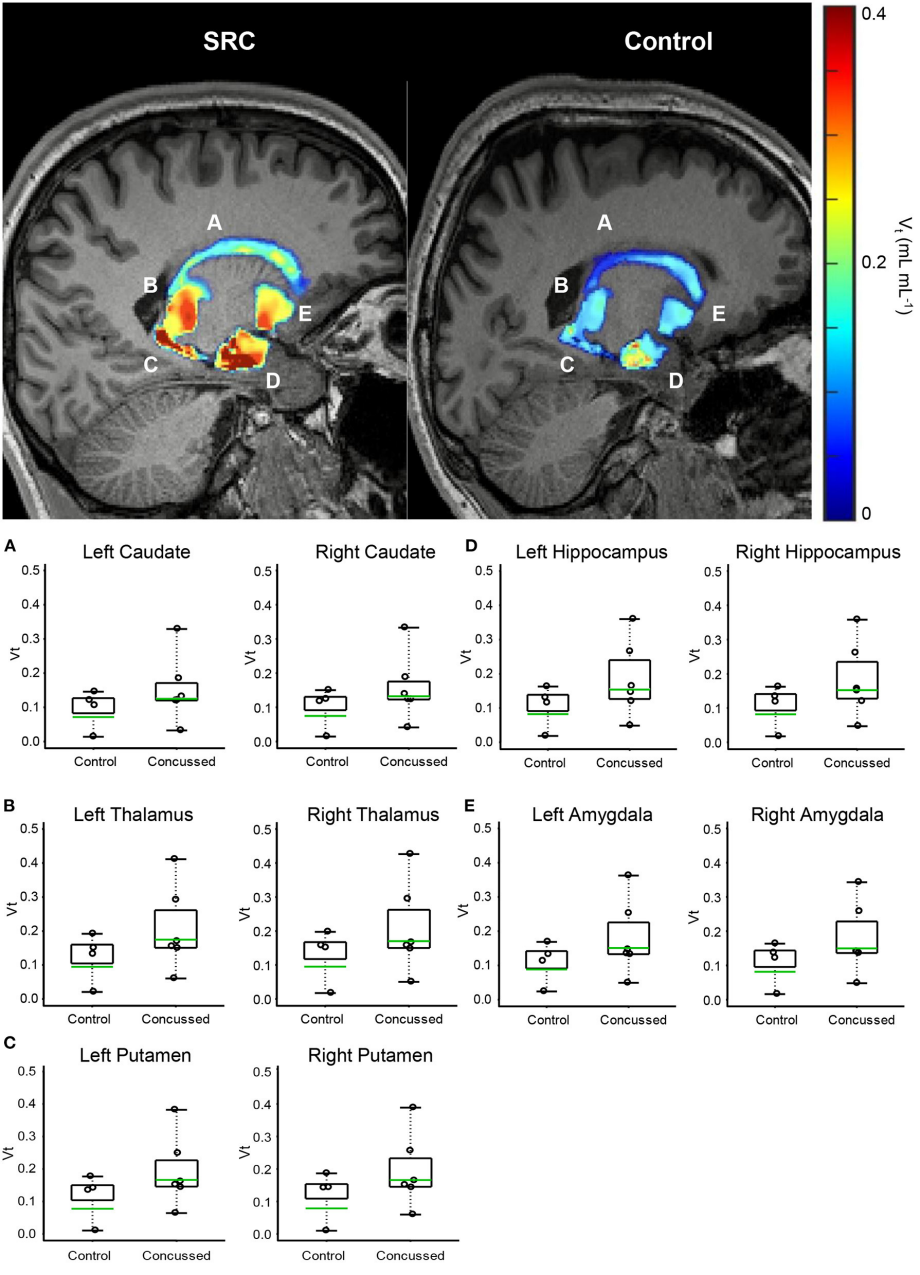
Volumetric VT maps quantify persistent microglial activation in athletes diagnosed with SC (Group 1) who have achieved uRTP compared to controls (Group 2). **(A)** Caudate. **(B)** Thalamus. **(C)** Putamen. **(D)** Hippocampus. **(E)** Amygdala. Regional VT maps are computed (top) from a voxel-based parametric PET map. Geometric means (bottom-green line) of voxels within each CNS region are compared (bottom) between SC athletes and age- and genotype-matched healthy controls. Across 164 regions, the regions associated with consistent and significantly elevated microglial activation are the bilateral caudate, thalamus, putamen, hippocampus, and amygdala.

Each voxel of the dynamic PET data was independently fed into a graphical Logan model (71) (written in Matlab) together with the MCIF. The model performed a linear regression on the data [starting at 10 min (72) and beyond for which the image data is linear], where the slope gives a measure of the total distribution volume, VT, at that voxel. The above method of computing MCIF is more quantitative as it accounts for PV recovery of the blood input. By analyzing millions of voxels across the entire brain PET volume, a parametric VT map was computed. The above masks (10 ROI's) generated in patient MR space were then dropped onto computed parametric PET map in Matlab (Mathworks Inc., Natick, MA) to obtain regional average VT (Figure 1). A schematic of the computational workflow is described in Supplementary Figure S1.

### Data analysis

For Group 1, repeated measures analyses of variance (ANOVA) were used to compare ImPACT (Verbal and Visual Memory, Visual Motor Speed, Reaction Time, and Total Symptom Score) and SOT (Equilibrium Score and Somatosensory, Visual, and Vestibular Sensory Ratios) outcome scores across Visits 1, 2, and 3. Effect size was calculated using partial eta squared (η^2^) with magnitudes interpreted as small (0.01–0.05), medium (0.06–0.13), or large (>0.14) (73). *Post-hoc* analyses were performed using paired *t*-tests when appropriate.

Between group comparisons were performed for the GAD-7, ImPACT, PHQ-9, PSQI, RAVLT, SCAT 5 (Total Symptom Severity, Orientation, Immediate Memory, Concentration, Delayed Memory), SOT, SWLS, and Trail Making Forms A and B using independent *t*-tests and Cohen's *d* to estimate effective size with 95% confidence intervals and interpreted as small (0.20–0.49), medium (0.50–0.73), or large (>0.80) (74). Specific to the ImPACT and SOT, Group 2's outcome scores from Visit 2 were compared to Group 1's outcome scores from Visits 1- 3. All analyses were performed with α = 0.05.

#### Brain region VT analyses

The VT measurements from brain regions were transformed to the natural logarithmic scale so that the comparison of the distribution of VT measurements from the athletes with SC (Group 1) and the healthy control non-athletes (Group 2) could be interpreted as a percentage difference $(i.e.,\;\;\frac{GM\;VT_{Group\;1\;-\;GM\;VT_{\;Group\;2}}}{GM\;VT_{\;Group\;2}}\;x\;100\%$) in the geometric mean (GM) of the VT measurement distribution.

#### Analytical methods

A Welch style *t*-test (75) was conducted per brain region to compare the geometric mean of the VT measurement distribution between athletes with SC and healthy controls. Per brain region, the null hypothesis assumed that the geometric mean ratio (i.e., Group 1:Group 2) is equal to 1, and the alternative hypothesis was that the geometric mean ratio is >1; i.e., higher VT for athletes with SC than for healthy control non-athletes. The Benjamin and Hochberg false discovery type I error rate procedure (76) was applied to the complete set of *p*-values derived from the 10 Welch *t*-test *p*-values.

Probability estimation for estimating the chance that all 10 brain regions would simultaneously produce a geometric mean (GM) VT ratio (i.e., GM VT _Group1_ : GM VT _Group2_) >1 was determined based on the binomial cumulative probability distribution, where under the null hypothesis it was assumed that for any particular brain region the probability (*p*) for the collegiate concussed athletes (Group 1) to have a GM VT greater than the GM VT of the healthy controls (Group 2) is 0.5 (i.e., *p*=1/2). The calculation further assumed that the GM VT ratios of the 10 brain regions are statistically independent.

## Results

### Human subjects enrolled

A total of 27 participants were recruited for this study. Participants were excluded due to being a low-affinity binder (*n* = 2), being diagnosed with a head injury 2 days prior to their scheduled MRI and PET scans (*n* = 1), did not complete the imaging protocol (*n* = 2), admitted to taking creatine (*n* =1), or they withdrew from the study (*n* = 4). One participant was excluded for creatine use, as recent evidence (33, 34) has suggested creatine use may confound TSPO PET signal (33, 77). Our final analyses included 8 (5 male, 3 female) active collegiate athletes. The athletes recruited for this study participated in a wide range of contact and non-contact sports including football, wrestling, cross country, diving, and rowing. Ten (5 male, 5 female) healthy collegiate students served as healthy controls. No significant differences were observed between groups for age or weight [*all p* > 0.05 (Supplementary Table S2)]. Participant breakdown by sport, concussion history, and the timing of the neuroimaging protocol may be found in Supplementary Table S3. All subjects who were not excluded from the study on the basis of rs6971 polymorphism (T/T genotype – LAB phenotype) completed both MRI and PET scans.

### Clinical assessment of NCAA athletes

Descriptive clinical data may be found in Tables 1, 2. Note, Group 2 only performed ImPACT and the SOT at Visit 2. Group 2's ImPACT and SOT data were compared to Group 1's Visit 2 and Visit 3 data. Table 2 represents descriptive data for the clinical measures administered during Visit 3 to both groups.

**Table 1:** A comparison of Groups 1 and 2 for the ImPACT battery and the SOT.

**Domain**	**Group 1 (*****n*** = **8)**			**Group 2 (*****n*** = **10)**		
	**Visit 1**	**Visit 2**	**Visit 3**	**Visit 1**	**Visit 2**	**Visit 3**
**ImPACT**						
Verbal memory	90.0 (9.57)	88.0 (10.86)	95.3 (4.11)	*NA*	97.8 (3.22)^†^	*NA*
Visual memory	78.4 (14.6)	72.4 (14.80)	78.4 (13.73)	*NA*	86.7 (10.30)^†^	*NA*
Visual motor speed	43.1 (5.18)	42.1 (6.25)	46.1 (4.51)	*NA*	46.8 (5.87)	*NA*
Reaction time	0.56 (0.06)	0.67 (0.13)	0.52 (0.04)^‡^	*NA*	0.53 (0.07)^†^	*NA*
Total symptom score	1.4 (1.81)	16.13 (15.13)	0 (0)	*NA*	0.7 (1.25)^†^	*NA*
**Sensory organization test**						
Equilibrium score	81.4 (3.93)	79.3 (3.69)	82.7 (5.56)	*NA*	83.9 (4.77)^†^	*NA*
Somatosensory ratio	96.1 (2.33)	96.8 (3.69)	97.3 (2.66)	*NA*	94.9 (3.43)	*NA*
Visual sensory ratio	93.0 (4.10)	92.1 (7.26)	94.9 (3.43)	*NA*	92.5 (5.56)	*NA*
Vestibular sensory ratio	73.2 (9.25)	75.3 (6.93)	76.5 (14.2)	*NA*	79.6 (5.04)	*NA*
**SCAT5**						
Symptom severity	*NA*	8.8 (7.17)	*NA*	*NA*	0.7 (0.95)^†^	*NA*
Immediate memory	*NA*	5.0 (0.0)	*NA*	*NA*	4.9 (0.32)	*NA*
Concentration	*NA*	22.8 (3.69)	*NA*	*NA*	24.8 (3.36)	*NA*
Delayed memory	*NA*	6.5 (2.00)	*NA*	*NA*	4.1 (1.0)	*NA*
Balance error scoring system	*NA*	4.3 (3.69)	*NA*	*NA*	1.8 (1.40)	*NA*

† represents a significant difference (*p* < 0.05) between Group 2's VISIT 2 and Group 1 performance at VISITS 2 or 3 for the ImPACT and SOT. It also represents a between group difference for the SCAT5 at VISIT 2.

‡ represents a significant main effect for across visits for Group 1 only.

**Table 2:** Descriptive data for patient reported outcome measures and neurocognitive assessments administered at VISIT 3.

**Domain**	**Group 1 (*n* = 8)**	**Group 2 (*n* = 10)**	***P-*value**	***d* (95% CI)**
**Patient reported outcome scores**				
GAD-7	0.38 (1.06)	2.6 (2.50)^†^	0.03	1.11 (0.09, 2.10)
PHQ-9	0.63 (0.92)	3.20 (1.25)	0.09	0.85 (−0.14, 1.81)
PSQI	6.5 (3.16)	5.0 (2.94)	0.31	−0.49 (−1.43, 0.46)
Satisfaction with life scale	28.9 (3.76)	30.0 (3.50)	0.52	0.31 (−0.65, 1.23)
**Neurocognitive testing**				
NIH flanker task	104.4 (14.65)	107.5 (5.02)	0.54	0.30 (−0.64, 1.23)
NIH oral reading recognition	111.8 (5.18)	115.2 (2.82)	0.09	0.86 (−0.13, 1.82)
NIH picture sequence memory	107.9 (20.10)	124.6 (15.10)^†^	0.06	0.96 (−0.39, 1.93)
Rey auditory verbal learning test	56.9 (8.44)	60.1 (6.17)	0.36	0.46 (−0.50, 1.38)
Rey auditory verbal learning test delayed	11.5 (2.93)	13.1 (2.02)	0.19	0.65 (−0.32, 1.60)
Trail making A	24.5 (10.13)	18.2 (3.51)	0.09	−0.87 (−1.83, 0.12)
Trail making B	48.6 (20.5)	41.2 (9.57)	0.32	−0.83 (−0.48, −1.42)
Word choice test	49.5 (0.93)	49.8 (0.42)	0.37	0.44 (−0.51, 1.37)

† represents a significant difference (*p* < 0.05) between groups.

#### GAD-7

At Visit 3, Group 2 endorsed significantly higher [*t*_(16)_ = 2.34, *p* = 0.02, *d* = 1.11 (0.090, 2.10)] levels of anxiety on the GAD-7 compared to Group 1 athletes with SC. However, there were no group differences in difficulty completing activities of daily living due to anxiety symptoms.

#### ImPACT

For Group 1, a significant main effect was observed only for the ImPACT Reaction Time score [*F*_(1.159, 6.954)_ = 7.59, *p* = 0.03, η^2^ = 0.56] with injured participants significantly faster at their symptom free assessment compared to their baseline [*t*_(16)_ = −2.25, *p* = 0.03] and Visit 2 assessments [*t*_(16)_ = 4.13, *p* = 0.003]. Symptom severity measures showed a significant main effect across time for the injured group [*F*_(1.018, 6.111)_ = 6.00, *p* = 0.049, η^2^ = 0.50]. Injured participants reported a significantly higher total symptom severity score at Visit 2 after SC compared to Visit 1 [*t*_(6)_ = 2.09, *p* = 0.04] and Visit 3 [*t*_(6)_ = 2.33, *p* = 0.03]. At Visit 2, Group 1 scored significantly lower on ImPACT's Verbal Memory [*t*_(16)_ = 2.74, *p* = 0.01, *d* = 1.29 (0.25, 2.31)], Visual Memory [*t*_(16)_ = 2.42, *p* = 0.01, *d* = 1.15 (0.12, 2.14)], and Reaction Time [*t*_(16)_ = −2.76, *p* = 0.01, *d* = −1.31 (−2.32, −0.26)] composite scores compared to Group 2. ImPACT's Total Symptom Score measures demonstrated a significantly higher symptom severity reported in Group 1 compared to Group 2 at Visit 1 [*t*_(16)_ = −3.24, *p* = 0.003, *d* = −1.53 (−2.59, −0.45)]. At Visit 3, no differences were observed between groups for any ImPACT outcome score (all *p* > 0.05). ImPACT's Total Symptom Score was similar between groups at the symptom free time point (*p* > 0.05).

#### NIH toolbox

At Visit 3, no significant differences were observed between Groups 1 and 2 on the NIH Flanker [*t*_(16)_=0.63, *p* = 0.27, *d* = 0.30], the Oral Reading Recognition [*t*_(16)_ = 1.81, *p* = 0.09, *d* = 0.86], and the Picture Sequence Memory [*t*_(16)_ = 2.02, *p* = 0.06, *d* = 0.96] tests.

#### PHQ-9

Participants in Group 2 self-endorsed significantly higher [*t*_(16)_ = 1.79, *p* = 0.046, *d* = 0.85 (−0.138, 1.81)] levels of symptoms of depression as compared to Group 1 at Visit 3. However, there were no group differences in difficulty completing activities of daily living due to these symptoms.

#### RAVLT

At Visit 3, no significant differences were observed between Groups 1 and 2 scoring on the RAVLT Immediate and Delayed Recall Composite scores (*p* > 0.05).

#### SCAT5

At Visit 2, Groups1 endorsed a significantly higher total symptom severity than Group 2 [*t*_(16)_ = 3.31, *p* = 0.002, *d* = 1.60, (0.57, 2.76)]. No significant differences were observed for Orientation, Immediate Memory, Concentration, Delayed Memory, or the Balance Error Scoring System within 24 h of a diagnosed SC.

#### SOT

Specific for Group 1, no differences were observed for any SOT outcome score (all *p* > 0.05). Our between group comparison for Visit 2 revealed Group 1 scored significantly lower [*t*_(16)_ = 2.232, *p* = 0.04, *d* = 1.06, (0.05, 2.04)] on the SOT Equilibrium Score compared to Group 2. No additional differences were observed for the remaining SOT outcome scores (all *p* > 0.05). At Visit 3, Group 1 performed similarly for all SOT outcome scores (all *p* > 0.05) to Group 2's Visit 2 performance.

At Visit 3, no differences were observed between groups for the PSQI Global score, the SWLS, or for Trail Making Test Forms A or B (all *p* > 0.05).

#### *In vivo* [^18^F]DPA-714 PET imaging

No significant differences between healthy student controls and NCAA Division 1 concussed athletes were noted with respect to injected dose of [^18^F]DPA-714, molar activity, and mass of [^19^F]DPA-714 as noted in Supplementary Table S2.

The advanced linear mixed model computing VT demonstrated elevated differences in the geometric means across all regions analyzed in Group 1 compared to Group 2. When normalized to genotype, C/T individuals (MAB) who were concussed and returned to play displayed anywhere from 71 to 115% increases in regional VT across 10 regions analyzed (Table 3), as well as a 25–41% higher regional VT among C/C (HAB) individuals (Supplementary Table S4). Predictably, individuals with C/C genotype displayed higher VT values when compared to those with the C/T genotype across all 10 ROIs tested. Participants in Group 1 displayed higher VT values than healthy controls in all 10 ROIs tested; Benjamin and Hochberg false discovery type I error rate procedure (76) did not reveal statistically significant differences when comparing populations means in individual regions. Although the threshold for statistical significance was not exceeded in any individual region, the chance of all 10 brain regions producing a Group 1: Group 2 VT geometric mean ratio greater than one was significant (*p* < 0.001). Representative PET images and individual data plots are also shown in Figure 1 and Supplementary Figure S2, respectively.

**Table 3:** Comparison of the distribution of VT between MAB concussed and MAB control subjects based on 2-sample Welch parametric *t*-test.

**Brain region**	**GM ratio _(Concussed:Control)_**	**Lower 95% CL**	**Upper 95% CL**	**% difference**	**Lower 95% CL**	**Upper 95% CL**	***P*-value**	**B&H threshold^†^**
Left hippocampus	1.87	0.43	8.20	87.06	−57.30	719	0.32	0.00833
Left thalamus	1.85	0.41	8.30	84.54	−58.96	729	0.33	0.01250
Left putamen	2.15	0.29	15.94	115.32	−70.91	1,493	0.34	0.01667
Right hippocampus	1.86	0.40	8.75	86.23	−60.35	774	0.34	0.02083
Left amygdala	1.71	0.46	6.43	71.28	−54.39	543	0.34	0.02500
Right amygdala	1.84	0.37	9.32	84.45	−63.50	832	0.36	0.02917
Right putamen	2.11	0.27	16.57	110.95	−73.15	1,557	0.36	0.03333
Right caudate	1.77	0.34	9.13	77.02	−65.68	813	0.40	0.04167
Right thalamus	1.79	0.33	9.55	78.55	−66.60	854	0.40	0.04583
Left caudate	1.74	0.34	8.82	74.15	−65.63	782	0.41	0.05000

† Note that if *p* ≤ B&H Threshold, the null hypothesis is rejected.

The Benjamini and Hochburg false discover threshold is listed for each comparison. Note that if the *p*-value is less than the B&H threshold^†^, the null hypothesis should be rejected with the assurance that the overall false discovery error rate (i.e., rate for rejecting the null hypothesis when the null hypothesis is true) for the complete set of null hypothesis tests will be no > 0.05 (i.e., 5%). GM, geometric mean.

## Discussion

It is increasingly understood that physiological recovery of SC may persist beyond clinical recovery based on current methods of clinical evaluation (5, 6, 17, 78–83). Our understanding of SC-related neuroinflammation is continually evolving based on these novel physiological measures. Despite these scientific advances, microglial activation is an important, yet poorly understood, physiological indicator of SC (84–89). Microglia are widely accepted as the resident immune cells of the central nervous system and they play an essential role in surveillance of brain parenchyma and homeostasis (18, 90–92). In the case of neuronal insult, such as mTBI, microglia transition from their surveilling state to a ramified state to promote both pro- and anti-inflammatory responses. Activated microglia have also been shown to influence synaptic remodeling and white matter recovery with brain injury (93, 94). Taken together, activated microglia, through PET-targeted TSPO overexpression, may serve as an important neuroimaging biomarker benchmark to inform future clinical measures and to identify therapeutic targets following SC.

In this study, we investigated whether microglial activation, as evidenced by [^18^F]DPA-714 binding in the brain, persists in collegiate athletes who reported symptom-free following a diagnosed SC. Importantly, all of the collegiate athletes participating in this study had been cleared for uRTP based on the current gold-standard clinical protocol, a graded exercise protocol, and a clinical examination (3, 4). Compared to healthy, age- and genotype-matched controls, all athletes demonstrated higher [^18^F]DPA-714 signal within the brain, particularly in the limbic system and medial temporal lobe structures despite scoring within normal limits on a multidimensional battery of tests in comparison to their baseline performance and a physically active collegiate student sample. Visual interpretation of [^18^F]DPA-714 images demonstrated striking differences between collegiate athletes who reported symptom-free following a medically diagnosed SC. Geometric mean comparisons of affected areas are also striking in that some athletes demonstrated higher changes in [^18^F]DPA-714 binding in upwards of 115%.

Although no individual region showed statistically significant differences in [^18^F]DPA-714 binding between groups, the results were notable in that every region tested trended toward higher binding in concussed athletes. *Post-hoc* statistical testing determined the chances of this finding were very unlikely (*p* < 0.001), if under the null hypothesis we assume for any particular brain region that the change for the VT geometric mean ratio to be greater than one is 0.50 and we also assume that the VT geometric mean ratios of 10 evaluated brain regions are statistically independent. Thus, this combination of results most likely reflects our small sample size and spatial variability of the brain regions affected in our concussed athletes. In addition, TSPO is known to be expressed on the vascular epithelium and astrocyte (95); thus, this nonspecific binding is expected to further dilute the subtle changes in VT observed in our limited sample size. Nonetheless, these differences in VT suggest a confirmatory study in a larger sample size is warranted.

TSPO expression in microglia, resulting from injury, has been well characterized in several preclinical models (85), which have shown a temporal dependency of PET imaging detection. In murine models of TBI, for example, [^18^F]DPA-714 binding is markedly higher between seven and 10 days post-injury, but contrast is lost ~2 weeks post-injury and beyond. The acute onset of microglial activation in athletes, as the result of a SC, in addition to interindividual variability of microglial activation, is not known. All collegiate athletes diagnosed with a SC in our sample underwent our neuroimaging protocol upon reporting symptom free and scoring within normal limits compared to baseline values standard-of-care clinical measures of SC. As previously mentioned, each participant in Group 1 made a full unrestricted return-to-play following a stepwise exercise protocol and clinical examination further indicating clinical recovery (2). The relative timeline for maximizing [^18^F]DPA-714 contrast, as a result of temporal changes in TSPO expression, is likely as variable as SC recovery is in each individual. Thus, our sampling timeline for imaging ranged from 8 to 30 days post-injury. Our single participant who underwent imaging at 8 days following injury did show higher [^18^F]DPA-714 binding in the temporal pole, however, binding within the medial temporal lobe and limbic system was not higher compared to the control cohort, which is contradictory to our results in the majority of other athletes enrolled in our study. Temporal trends of TSPO status may be more suited to quantify and predict functional recovery of an athlete and should be studied further in a larger cohort of subjects to confirm these changes in microglial activation status. In addition, a larger study with important controls for sub-concussive impacts and a concussion-negative, orthopedic injury are warranted.

This study represents an important first step in understanding central microglial activation associated with SC and the relative timeline these phenotypic changes occur within and beyond clinical recovery. This is an important distinction from previous studies examining TSPO status in athletes participating in collision sports (33, 34, 89). Coughlin et al. reported [^11^C]DPA-713 binding was significantly higher in active and retired NFL athletes compared to healthy controls. While this was a major finding and spurs many important research questions, these athletes were “late-stage”, i.e.,—career athletes participating in collision sports, compared to the athletes in our study. In fact, many of the participants in Group 1 of our study did not have a prior history of concussion. Thus, it is likely microglial activation in NFL athletes represents a more chronically activated state of microglia (58, 96), which may or may not be related to repetitive head impacts (97). For example, preclinical evidence demonstrates pro-inflammatory and anti-inflammatory microglia are upregulated (higher) following repeated mTBI (one injury every hour for 4 h) within 72 h of injury; however, a predominance of the pro-inflammatory phenotype was observed among the total microglial population (98). In addition, the majority of pro-inflammatory markers were resolved within 72 h following only a single concussive impact, thus, the cumulative pathologic response clearly has a longer temporal resolution course. Furthermore, one must consider ligand-target interactions of the imaging agents, irrespective of pharmacological dose (or lack thereof, in the case of PET) being administered. DPA-714 is an agonist, whereas DPA-713 is an antagonist, and agonists traditionally bind to high affinity states of a given receptor. Affinity state of TSPO has been suggested to be linked to neuroprotective vs. neurodegenerative activity of microglia (99), where chronically activated microglia may contain more TSPO in the high affinity state (99). Thus, if confirmed, [^18^F]DPA-714 binding may represent an important early distinction of pro-inflammatory microglial status in an athlete following SC altogether.

An additional aspect of this study that should be stressed is that all injured collegiate athlete participants returned to pre-injury values on clinical measures including balance, neurocognitive function, symptoms, and clinical examination (2, 6). Our findings are similar of those that have demonstrated persisting deficits using global connectivity, cerebral blood flow, fractional anisotropy, and mean diffusivity protocols in high school and collegiate athletes (6, 83). In alignment with this body of research, we observed persisting microglial activation in athletes, despite their return to normal limits on a comprehensive clinical evaluation. Our results cannot and should not be interpreted as indicating the onset of chronic microglial activation as suggested by other research in this area, as our findings may also reflect a normal physiological response to SC. Rather, these data are an important first step in understanding the true physiological response and recovery from SC in collegiate athletes.

Unique to our findings are a substantially higher PET signal indicative of persisting microglial activation for two injured participants (Table 3). Of note, these two participants were both female and had a prior history of three concussions. Only three other participants in Group 1 had a history of one or two concussions, but their microglial activation was similar to that of those with no history of concussion. It is important to note that, at this point in the emerging body of research related to central microglial activation using [^18^F]DPA-714, it is unknown whether this enhanced binding is associated with biological sex, concussion history, or if this enhanced neuroinflammatory response is neuroprotective or neurotoxic. That said, a history of one or multiple concussions has been associated with increased susceptibility for subsequent injury after uRTP (100, 101), increased risk of depression following retirement from sport (11), and additional general health and neurodegenerative conditions in mid- to late-life (102). However, a causal link for each of these poor outcomes following one or more concussions has yet to be established. For example, three or more self-reported concussions has been associated with less favorable outcomes, however, the underlying mechanism for this increased susceptibility is not understood (100, 102). Caution is warranted when inferring any association between the findings of this study provided its limited sample size and considerable limitations. It is important to note that while [^18^F]DPA-714 has been well documented to be useful in other neurodegenerative considerations, the extent of the role of additional mitigating factors (e.g., biological sex, age, physical fitness, and other variables) have yet to be fully understood. Substantial research is needed to confirm and extend the findings of this pilot study.

One limitation to this study is that metabolite corrected input functions were not sampled from the radial artery over the entirety of the scan. While this is the gold standard for calculating the MCIF (and subsequent VT), arterial catheterization and repeated sampling comes with inherent practical recruitment challenges and safety concerns to the study participant. Anecdotally, participants may be hesitant to participate in studies requiring arterial catheterization, particularly athletes. Instead, we computed the MCIF using an image-derived iteration that measures radioactivity per volume within the carotid arteries over the 90-min scan (52). These data were then standardized to a known radioactivity/volume of blood from four late and evenly spaced sampling points (45, 60, 75, and 90 min) as described above for MCIF computation (52). In addition, our calculated parent:metabolite ratios from each venous blood sample and extent of metabolism of [^18^F]DPA-714 did not significantly differ among group participants and were also in agreement with previously reported human data (72, 99). Taken together, these differences are not expected to have a meaningful impact in comparing VT differences between each cohort. Another important limitation is that our control group consisted of healthy, active collegiate students who were not part of a formal collegiate athletics team. Though the selection of participants in Groups 1 and 2 are similar to recruitment strategies related research (33, 34), confirmation and extension of our findings with matched, healthy, non-concussed controls from the same sport and non-contact sports is needed. It is possible that the intense physical activity, the time constraints, stress, and the associated negative impact on sleep may have also contributed to the neuroinflammatory response in these collegiate athletes. The research team elected to administer the clinical measures in alignment with the university concussion protocol at limited time points to minimize undue burden on symptomatic athletes.

In summary, our study is the first to demonstrate persistent microglial activation using [^18^F]DPA-714 in concussed athletes, following a battery of clinical measures, completing a clinical examination, and progressing through a stepwise exercise protocol allowing uRTP to their respective sport. Our findings further our understanding of the functional recovery following SC in collegiate athletes. However, our results are not conclusive as to whether the observed microglial activation in these subjects is the normal course of pro-inflammatory injury resolution or the onset of an anti-inflammatory chronic pathology (33, 34). As it is increasingly understood that an incongruence exists between clinical and physiological recovery from SC (5, 6, 83), these data are foundational toward understanding the presence of microglial activation in athletes following SC.

## Data availability statement

The original contributions presented in the study are included in the article/Supplementary material, further inquiries can be directed to the corresponding author.

## Ethics statement

The studies involving human participants were reviewed and approved by the University of Virginia Institutional Review Board and the University of Virginia Radioactive Drug Research Committee. The patients/participants provided their written informed consent to participate in this study.

## Author contributions

KN, DB, BK, and JER conceived the experiments. KN, JD, XT, JR, BK, and JER performed the experiments. KN, VS, JD, JM, BN, XT, JP, BK, and JER analyzed the data. KN, BK, and JER wrote the paper and all authors reviewed and edited. KM contributed to the radiosynthesis of [^18^]FDPA-714, as well as conducting sample perpetration and HPLC analysis of the blood samples taken at the various time points. All authors contributed to the article and approved the submitted version.
