# Supplementary material for: Microglial activation persists beyond clinical recovery following sport concussion in collegiate athletes
Source: Front Neurol. 2023 Mar 24;14:1127708. doi: 10.3389/fneur.2023.1127708 (PMC10080132; doi:10.3389/fneur.2023.1127708)

Supplementary Material

Neuroinflammation Persists Beyond Clinical Recovery Following Sport Concussion in Collegiate Athletes

Kiel D. Neumann, Vikram Seshadri, Xavier D. Thompson, Donna K. Broshek, Jason Druzgal, James C. Massey, Benjamin Newman, Jose Reyes, Spenser R. Simpson, Katelyenn S. McCauley, James Patrie, James Stone, Bijoy K. Kundu, Jacob E. Resch*

*** Correspondence:** Corresponding Author: jer6x@virginia.edu

Supplementary Figures and Tables

Table S1. A summary of clinical and physiological measures performed at each visit for groups 1 (injured athletes) and 2 (healthy controls). For Group 2, the (*) indicates clinical measures used for comparison to Group 1's outcome scores at VISITS 1-3

		VISIT 1 (Baseline)	VISIT 2 (24-hour Assessment)	VISIT 3 (Symptom Free Assessment)
**Group 1**	**Clinical Measures**	ImPACT<br>SOT<br>HIS-r	ImPACT<br>SOT<br>SCAT5	GAD-7<br>ImPACT<br>NIH Toolbox<br>PHQ-9<br>Rey Auditory Verbal Learning Test<br>SOT<br>Trail Making A and B<br>Word Choice Test
	**Physiological Measures**		Genotyping	Neuroimaging Protocol
**Group 2**	**Clinical Measures**	*NA*	ImPACT*<br>SOT*<br>SCAT5	GAD-7<br>NIH Toolbox<br>PHQ-9<br>Rey Auditory Verbal Learning Test<br>Trail Making A and B<br>Word Choice Test
	**Physiological Measures**	*NA*	Genotyping	Neuroimaging Protocol

Table S2. Participant clinical, demographic, and radiopharmaceutical injection characteristics. Characteristics of Enrolled Subjects. Abbreviations: C/C, high affinity binder; C/T, mixed affinity binder. **P* values for *t* test or Fisher exact test were calculated as appropriate. Threshold for significance, $P < .05$.

**Demographic Variable**	**Injured (*n* = 8)**	**Group 2 (*n*=10)**	***P***
Age (mean[SD])	19.6 (1.2)	21.6 (2.7)	0.06
Mass (kg) (mean[SD)	82.0 (28.6)	80.0 (14.6)	0.68
Rs6971 TSPO genotype (*n)*			
C/C (High affinity binders)	4	2	
C/T (Mixed affinity binders)	6	6	0.88
Injected dose (MBq), (mean[SD])	289(89)	277(30)	0.37
	31.4(56.0)	28.3 (29.0)	0.51

Table S3. Characteristics of 8 NCAA Division 1 athletes and 10 healthy student controls.

	Age	Biological Sex	Sport	Days post-concussion*	Known prior concussions
**NCAA Athletes**	21	Male	Wrestling	23	1
	18	Male	Football	26	0
	19	Male	Football	8	1
	21	Male	Diving	21	2
	18	Male	Track and field	30	0
	20	Female	Rowing	20	3
	20	Female	Cross Country	29	3
	20	Female	Rowing	27	0
**Healthy Student Controls**	22	Male		N/A	0
	21	Male		N/A	0
	21	Male		N/A	0
	22	Male		N/A	0
	29	Male		N/A	0
	20	Female		N/A	0
	20	Female		N/A	0
	21	Female		N/A	0
	21	Female		N/A	0
	20	Female		N/A	0

*Days post-concussion indicates the time lapse between diagnosis of injury by an athletic trainer and physician and the time of PET scan.

Table S4. Comparison of distribution of VT between HAB concussed and HAB control subjects. GM = geometric mean.

	**GM Ratio** (Concussed : Control)	**% Difference**
**Left Hippocampus**	0.810	41.2
**Left Thalamus**	0.812	40.5
**Left Putamen**	0.823	34.0
**Right Hippocampus**	0.812	35.7
**Left Amygdala**	0.817	40.1
**Right Amygdala**	0.813	40.9
**Right Putamen**	0.886	24.8
**Right Caudate**	0.848	34.1
**Right Thalamus**	0.819	38.4
**Left Caudate**	0.813	39.7

Figure S1. Image Processing, modeling and analysis pipeline. We have developed a robust advanced image analytics workflow to compute VT at the voxel level over the entire brain volume.

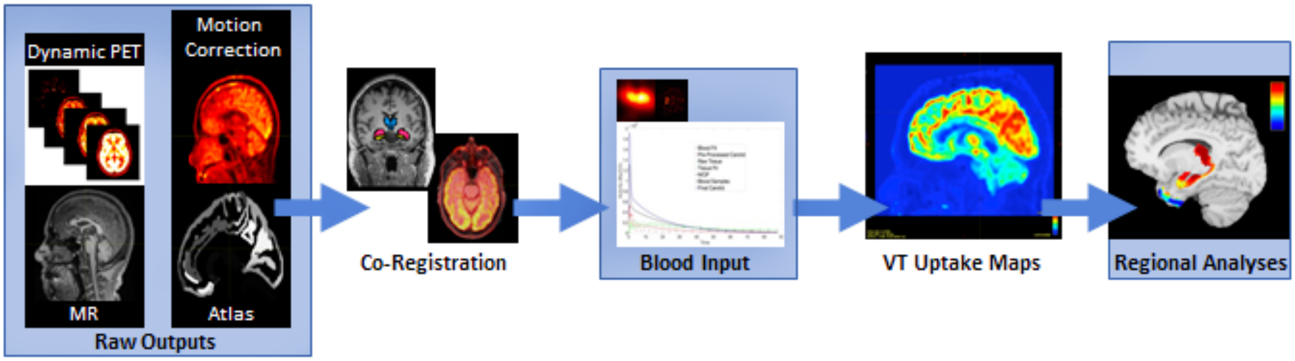

Figure S2. Boxplot displaying the VT distribution within each CNS region as compared between athletes with SC (right) and healthy age- and genotype-matched controls (left).

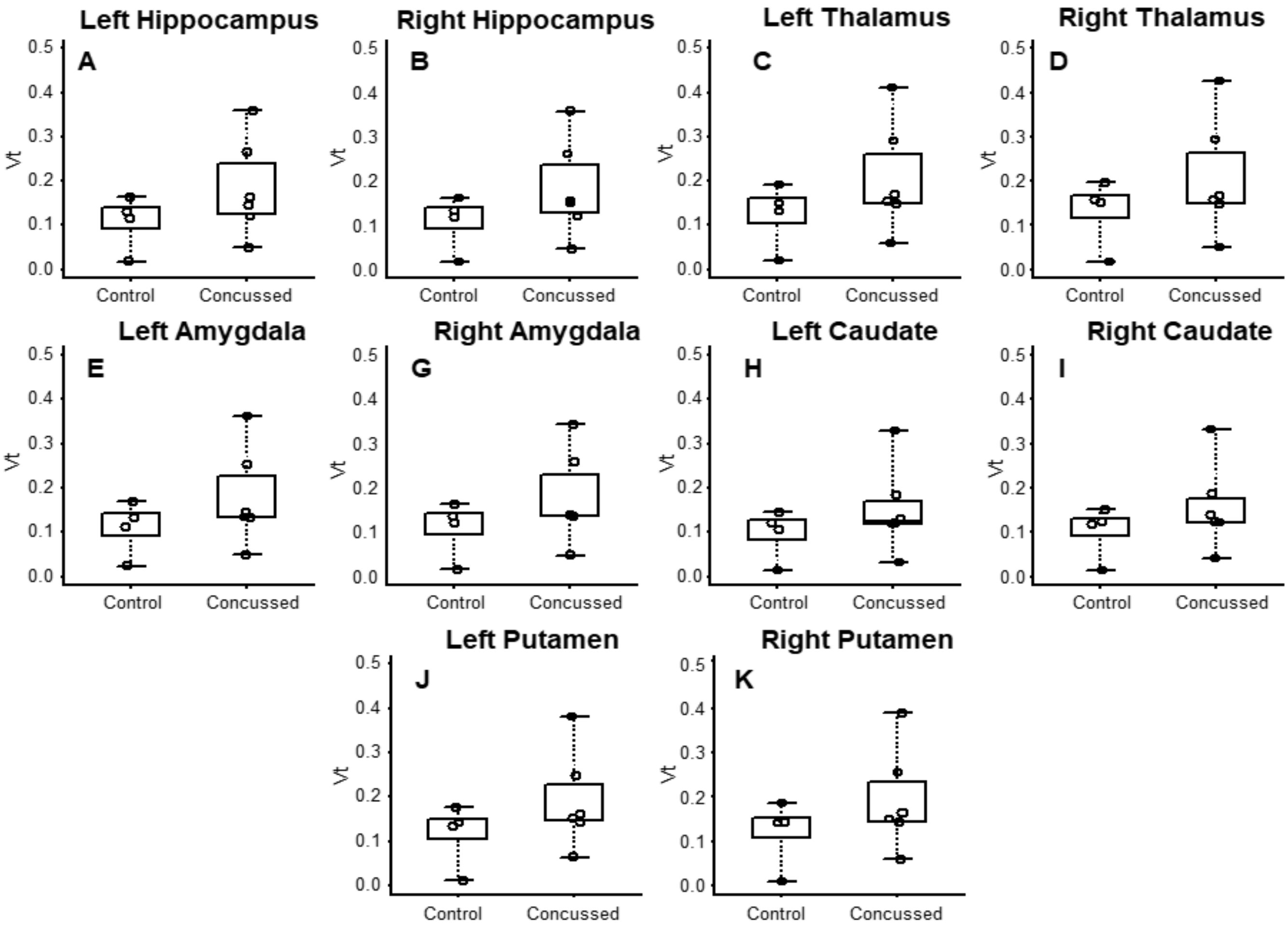

Supplement: Supplementary file 1 [file Data_Sheet_1.PDF]
